# Supplementary material for: Clinical trial and detection of SARS-CoV-2 by a commercial breath analysis test based on Terahertz technology
Source: PLoS One. 2022 Sep 20;17(9):e0273506. doi: 10.1371/journal.pone.0273506 (PMC9488804; doi:10.1371/journal.pone.0273506)
Supplement: S1 File — (PDF) [file pone.0273506.s002.pdf]

Clinical trial and detection of SARS-CoV-2 by a commercial breath analysis test based on Terahertz technology

Introduction

The outbreak of the new Coronavirus, initially named 2019-nCov, thus described in Wuhan, China, and now known as SARS-CoV-2, resulted in the declaration of a pandemic status by the WHO on March 11, 2020(1). The exceptional and unprecedented situation determined by the pandemic resulted in major social transformations, with an enormous (immediate and late) impact on public and private health worldwide. In Brazil, the first case was detected on February 25, 2020 and the government established measures to prevent and control COVID-19, following epidemiological, sanitary and legal criteria (2).

It is uncertain what the future pattern of COVID-19 activity will be, but there is certainly a big impact for Brazil (2). However, it is right to analyze the dynamic character of evolution, as well as differences in its regional behavior in our continental country, not to mention the huge regional differences in the quality of public and private health services. The distribution of the health crisis is not homogeneous and simultaneous across the planet; in Brazil, a country of continental proportions, the distribution is unequal as is the period of peaks of viral infection. In this sense, the public authorities have a fundamental role in the permanent assessment of the geographic behavior of the spread of COVID-19. On the other hand, the pandemic did not prevent the natural evolution of conditions unrelated to the new Coronavirus.

Healthcare systems around the world are challenged like never before in history. A dilemma arises: how to return to daily life without causing further complications? It is essential to point out that the resumption in an unstructured way can cause an increase in mortality (8-10) (11).

One possible way is to seek rapid, low-cost and highly effective diagnostic methods. If it is possible to find a method that can do mass screening, it would be possible to safely resume activities. Among the possibilities put forward, are diagnostic methods through blowing equipment, similar to breathalyzers, which can directly detect the presence or affirm the inexistence of the SARS-CoV-2 virus. The world literature points to a vague literature of methods under development for the analysis of VOC (Volatile Organic Chemicals) for the diagnosis of pneumonia caused by different pathogens (3-7).

Tera Group LTD from Israel has developed initial research in the area of diagnosis of SARS-CoV-2 through Breath Analyzes with promising results according to preliminary results.

After an international agreement between TERA of Israel and the State of Paraná, a protocol was proposed to analyze the effectiveness of the technology in a prospective way, according to the attached protocol.

TERA test preliminary results

Analysis of 801 patients with 175 positive for COVID-19 in RT PCR and 626 negative showed promising results with an ROC curve with an area of 0.765 as shown in the figure below.

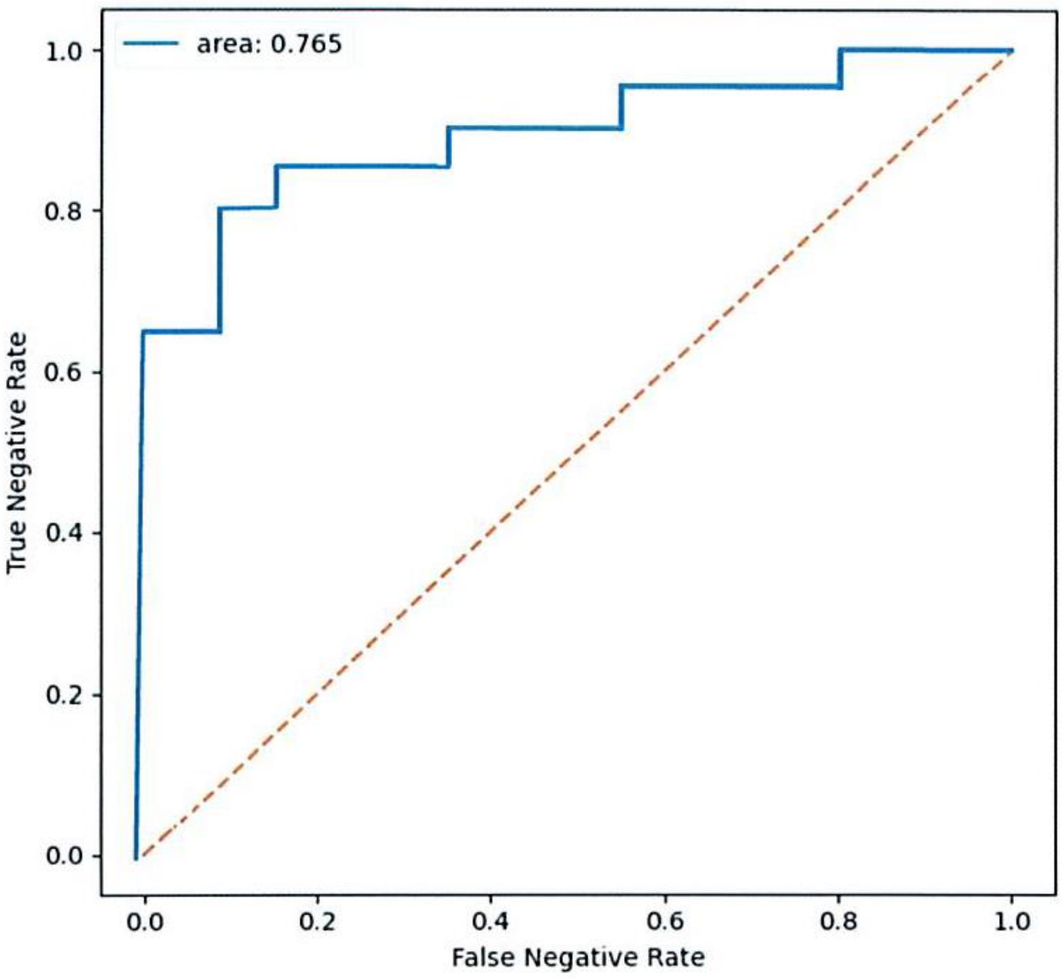

These initial findings allowed the construction of sensitivity and specificity simulation curves as follows:

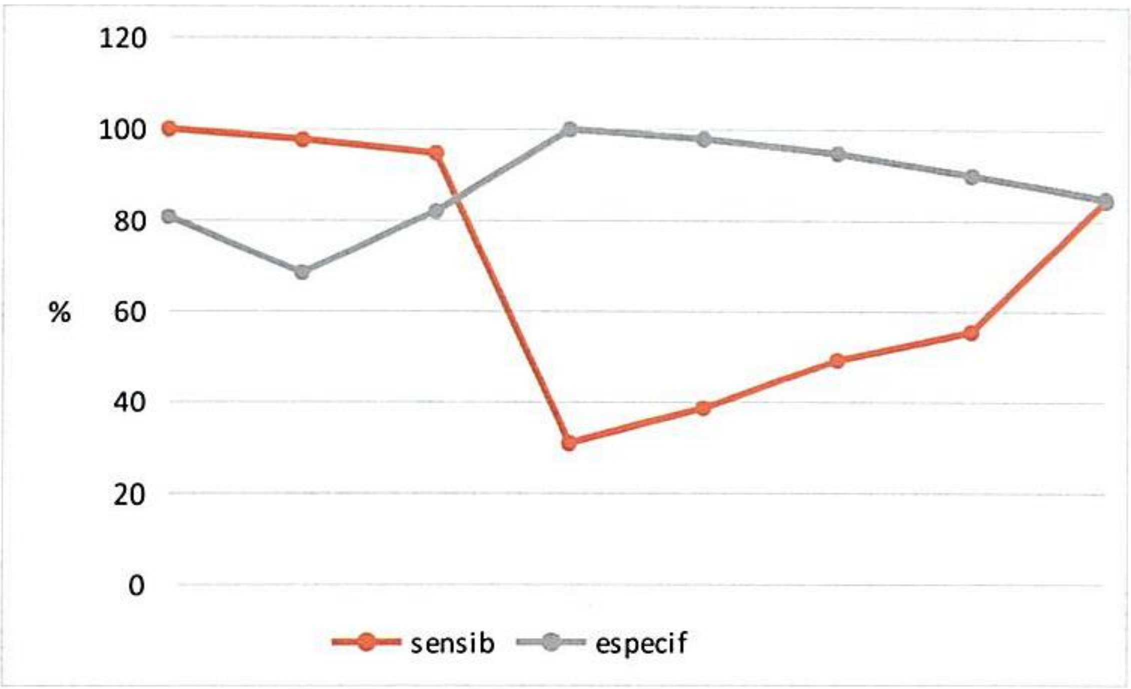

It is noticed that the greatest potential is probably linked to the ability to exclude cases, that is, negative predictive value, which is associated with high specificity.

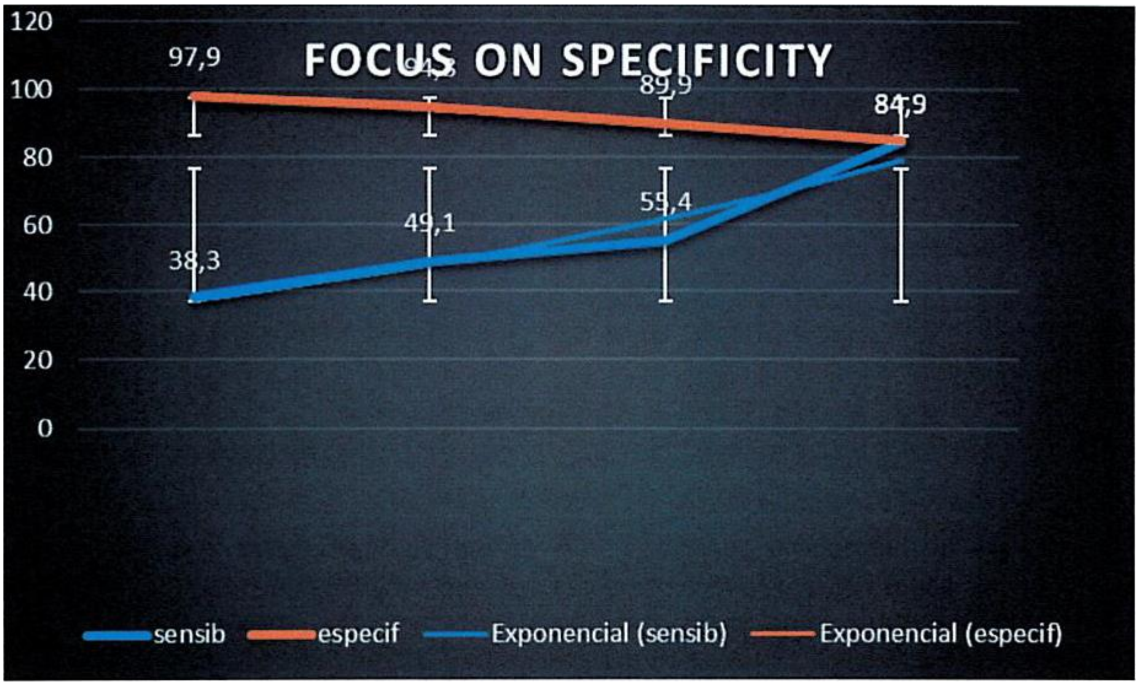

Objectives

This research will establish the preliminary database for a quick feasibility study. If it is found that the virus can indeed be identified (True Positive), or cases ruled out (True Negative), the research should be scaled up to allow for the rapid development of screening tests for SARS-CoV-2.

Primary Objective:

Development of a fast, cheap and assertive test to rule out SARS-CoV-2 in expired air.

Methods

Detection of the presence of SARS-CoV-2 VOC (Volatile Organic Chemicals) in samples collected from patients tested by spectral characteristics in the THz band in samples of expired air on the membrane of a respiratory test tube. The spectral analysis is done in the spectrum of 300-3000 GHz.

The RT-PCR will be performed by LACEN or IBMP and according to the current protocol; it will be considered the gold standard comparator. The group that will perform the PCR will be blind to the Tera results. Technicians from Tera Group, which owns the technology, and will be blind to the PCR result, will perform the BAT – Breath Analysis Test. Tecpar principal researcher and its group will compile and analyze the data.

If the PCR is positive and the BAT is negative, the BAT should be repeated as soon as the PCR result is available. If the BAR is positive and the PCR is negative, the PCR should be repeated as soon as possible. Any repetition will be performed in the same places described

above, using the same technique. The researchers and the company TERA Brasil will incur additional unusual costs.

If both BAT and PCR are concordant (negative or positive), no test needs to be repeated.

Statistical analysis

There will be a sensitivity and specificity analysis performed by the traditional 2x2 cross table and correlation analysis to be presented by ROC analysis and correlation coefficient determination.

There will be analysis considering 2 groups of patients together or separately, considering the presence or absence of signs and symptoms. Patients should be analyzed as follows:

1. Group 1.1 – Positive symptomatic PCR
2. Group 1.2 – Negative symptomatic PCR
3. Group 2.1 – Positive asymptomatic PCR
4. Group 2.2 – Negative asymptomatic PCR

The subanalysis is planned considering asymptomatic patients without previous exposure.

**Test/Day**	**D0**	**D0**	**D0**	**D0**
**PCR**	**Negative**	**Positive**	**Positive**	**Negative**
**BAT**	**Negative**	**Positive**	**Negative**	**Positive**
**Action**	**No**	**No**	**Repeat BAT**	**Repeat PCR**

Number of patients to be tested:

The study is expected to include a minimum of 1,500 test subjects verified as COVID-19 carriers, and a minimum of 1,500 subjects verified as non-carriers.

However, there will be an interim analysis when 100 positive PCRs are included. This analysis will provide additional data to adjust the total number of patients to be tested.

Inclusion Criteria:

Male and female

Age ≥18 years old

The patient must accept and sign the informed consent form.

Carrier status of the SARS-CoV-2 virus will be determined on a sample collected at the same time as the breath analysis test (BAT).

Outpatient cases

Symptomatic and asymptomatic.

Exclusion Criteria:

Hospitalized patients

Subjects under general anesthesia

The inability to sign the consent form personally

The inability to blow 5 times on the test

BAT samples analyzed after 6 hours.
Inability to have a second sample
Symptomatic patients with more than 5 days of symptoms for the first PCR sample or more than 7 days for repeat

Acquisition Strategy

The objective is to capture patients who are currently treated at Osvaldo Cruz Hospital by Drive Thru and may be considered other patients treated in the public and private system of RT-PCR exams for symptomatic patients and their contacts. Therefore, patients who would already undergo the RT-PCR test because they have a diagnostic suspicion will be invited at the time of requesting and/or performing the exam, to participate as described in the TCLE. Likewise, on this contact, it will be explained that the exam can be repeated in case of doubtful results, which is already done habitually. At this time, the researchers can also invite the patient to offer the test to their family members, both symptomatic and asymptomatic. In this case, the researchers will make telephone contact directly with the patient. Patients will also be informed that the RT-PCR results (first sample and eventual repetitions) follow the same usual operational flow, with results available in the official reporting system. On the other hand, the results of the breath tests (VOC) will be informed only after the research is completed and will be done by the researchers in an active way and with direct contact with the patients.

Test Procedure

A trained authorized member will explain to the tested subject about both tests (BAT and PCR) and will give you the informed consent form.

A researcher/research assistant or other staff member will give an explanation to the patient – after having been instructed and certified by the lead researcher, and explicit consent from the patient. The explanation must be made verbally or by filling out an online form (to avoid contact with the patient). The researcher must document the patient's consent and encode the patient's name with a unique identification. The lead researcher must keep the encryption key.

a. The form with the information collected must be labeled with a sticker containing the test person's unique ID if the electronic record is not running at that time.
b. The tested subject will review and sign the consent form and return it to the team member.
c. Subsequently, the membrane is extracted from the tube and placed inside the capsule to be scanned by the TeraSystem for analysis of its biochemical spectral signature within the THz range.
d. Samples collected from different locations must be in place for BAT analysis in less than 6 hours.
e. Transport must be carried out using regulatory biosafety rules. There is no need for temperature control throughout the process.

The breathalyzer test procedure and medical information will be handled as follows:

Step 1 – A subject blows 5 times into the disposable tube, a portable plastic test tube (provided with the membrane). The constituents of the aerosol breath are then collected on the sterile membrane.

Step 2 – The membrane is extracted from the tube and transferred to a sterile disposable capsule. Teflon capsule will be used.

Step 3 – The sterile capsule is then scanned with the TeraSystem by a technician.

Step 4 – All scanning processes are being monitored by a test technician.

Step 5 – During the validation phase, the anonymous subject data (from the subject questionnaire, section below) will be incorporated into the subject information form by the administrative assistants.

**Minimum patient data**
Age:
Gender: Male/Female
Height (ft/cm):
Weight (lbs/kg):
Tobacco use:
Date of first symptom:
Medications in use:
Chronic Illnesses/Background Illnesses:

Step 6 – At the end of the workday, the collected anonymous data will be sent (via the internet) to BioSafety Technologies/Teragroup servers, subject to BioSafety Technologies' personal approval.

Step 7 – The collected anonymous data will be analyzed.

Step 8 – Biosafety technologies team will provide BAT results to the project's medical center.

Step 9 – TECPAR will have the SARS-CoV-2 RT-PCR result and define the conduct with each case (other respiratory virus found in samples will be analyzed later).

Procedures from the investigators point of view.

1. The information form and the tested subject will be co-assigned through a unique code (QR/Barcode).
2. All breath samples and breath analysis kit parts used by a patient are considered contaminated until disinfected (by ethanol, autoclave or other medically acceptable disinfection method) and will only be handled by a certified/protected hospital/test site staff member.
3. The following part of the protocol will be performed by a trained local authorized employee, within the testing site:
    a. The subject will blow 5 times into the test tube containing the membrane.
    b. The subject or team member will extract the membrane from the tube, enclose it inside the capsule, and seal it.
    c. The team member will check the capsule seal.

d. The team member will place the sealed tested capsule into the biohazard bag, seal the bag, and place it in a biohazard container with all other sample capsules.
e. The plastic breathing tube will be handled separately (see section H below).
f. Upon completion of sample collection at the test site, and no later than six (6) hours from the time of sample collection, the following steps must be taken:
   A. All capsules that will be collected within the breath sampling site will be placed in a biohazard box, which the team member will carry when leaving the testing room.
   B. A 70% ethanol bath will be placed outside the complex.
   C. The employee will take the sealed capsules out of the biohazard bags and immerse them in the 70% ethane bath for at least 15 minutes.
   D. The team member will remove the capsules with designated clean tweezers, after ensuring that all tags remain on the capsules.
   E. Sterile capsules will be transferred for immediate scanning at test sites.
   F. Upon completion of the spectrum reading of the samples, the used capsules (containing the contaminated membrane) will be dumped in the biological waste bin within the designated location.
   G. The team member will take the used tubes out of the biohazard bags and immerse them in the bath of ethanol for 15 minutes.
   H. The employee will transfer the disinfected tubes from the ethanol for further cleaning and autoclaving (up to 1200°C) allowing for reuse.
   I. Locating the sweep sampling room in a separate clean area outside the breath-sampling site will allow for immediate examination after disinfection of the capsules, meeting the target of a test within a short space of two hours.

Procedures from the patients' point of view

As it is a non-invasive method, no side effects are expected. It is possible that the patient experiences some discomfort related to the collection of the nasopharyngeal swab of the RT-PCR test, which should be performed together with the breath, noting that this test would be done anyway because it is the gold standard and operational routine of the service.

The risk related to the study is that the result demonstrates insufficient sensitivity and specificity for the diagnosis of COVID-19, causing the test to be unfeasible.

The expected benefits of this research are rapid, inexpensive, non-invasive and assertive diagnosis to rule out SARS-CoV-2 in expired air. By carrying out the validation and registration, the exams can be performed at various points in the public and private sector.

Simplified Schedule

ACTION	DATE OR TERM	NOTE
PROTOCOL REVIEW	JULY 01, 2020	Concluded
SUBMISSION TO CEP	JULY 10, 2020	Pending

**ISRAEL EQUIPMENT AND TECHNICIANS**	**3 TO 5 DAYS**	**AFTER APPROVAL OF CEP**
**START OF THE PROJECT**	**1 TO 2 DAYS**	**AFTER INSTALLATION**
**DATA COLLECTION**	**15 TO 20 DAYS**	**AFTER COLLECTION OF 100 POSITIVE PCR**
**INTERIM ANALYSIS**	**1 TO 3 DAYS**	
**NEXT STEPS AND DEFINITIONS MEETING**	**1 TO 3 DAYS**	**PROBABLE DATE AUGUST 21 TO 31, 2020**

Financing

The researchers and TERA Brasil will subsidize all additional costs.

ANNEXES

Annex 01 – Schedule

Annex 02 – Budget

REFERENCES

1. Clinical management of severe acute respiratory infection when COVID-19 is suspected. Available from: https://www.who.int/publications-detail/clinical-management-f-severe-acute-repiratory-infection-when-novel-coronavirus-(ncov)-infection-is-suspecte
2. Cimerman S, Chebabo A, Cunha CA da, Rodriguez-Morales AJ. Deep Impact f COVID-18 in the healthcare of Latin America: the case of Brazil. Brazilian J Infect Dis (Internet). 2020 Apr [cited 2020 Apr 27]; Available from: https://linkinghub.elsevier.com/retrieve/pii/S1413867020300325
3. PM van O, P, P, R, S, P D, A, A, DCJJ B, et al. The Potential Role of Exhaled Breath Analysis in the Diagnostic Process of Pneumonia-A Systematic Review. J Breath [Internet]. 2018 [cited 2929 Jul 10];12(2). Available from: https://pubmed.ncbi.nlm.gob/29292698/
4. J G, Y Z, F W, L L, PQ, et al. Breath Analysis for Noninvasively Differentiating Acinetobacter Baumanni Ventilator-Associated Peneumonia From Its Respiratory Tract Colonization of Ventilated Pacients. J Breath Res [Internet]. 2016 [cited 2020 Jul 10];10(2). Available from: https://pubmed.ncbi.nlm.gov/27272697/
5. van OortPMP, Nijsen T, Weda H, Knobem H, Dark P, Felton T, et al. BreathDx – Molecular analysis of exhaled breath as a diagnostic test for ventilator-associated pneumonia: Protocol for a European multicenter observational study. BMC Pulm Med [Internet]. 2017 Jan 3 [cited 2020 Jul 10]/17(1). Available from https://pubmed.ncbi.nlm.nih.gov/28049457/
6. Schnabel R, Fijten R, Smolinska A, Dallinga J, Boumans ML, Stobberingh E, et al. Analysis of volatile organic compounds in exhaled breath to diagnose ventilator-associated pneumonia. Sci Rep [Intenet]. 2015 Nov 26 [cited 2020 Jul 10];5. Available from: https://pubmed.ncbi.nlm.nih.gov/26608483

7. Cao B, Wang Y, Wen D, Liu W, Wang J, Fan G, et al. A Trial of Lopinavir-Ritonavir in Adults Hospitalizaed with Severe Covid-19. N Engl J Med. 2020;
8. Coccolini F, Perrone G, Chiarugi M, Di Marzo F, Ansaloni L, Scandroglio I, et al. Surgery in COVID-19 patients: operational directives. World J Emerg Surg [Internet]. 2020 Apr 7 [cited 2020 Apr 20];15(1):25. Available from: https://ncbi.nlm.nih.gov/pubmed/32264898
9. Clinical Issues and Guidance [Internet]. [cited 2020 Apr 20]. Available from: http://www.facts.org/covid-19/clinical-guidance
10. Fang L, Karakiulakis G, Roth M, Li Q, Guan X, Wu P, et al. Early Transmission Dynamics in Wuhan, China, of Novel Coronavirus-infected Pneumonia. Lancet Respir Med [Internet]. 2020 [cited 2020 Apr 11]; Available from: https://www.idsociety.org/practice-guideline/covid-19-guideline-treatment-and-management/
11. Lei S, Jiang F, Su W, Chen C, Chen J, Mei W, et al. Clinical characteristics and outcomes of patients undergoing surgeries during incubation period of COVIF-19 infection. EClinicalMedicine [Internet]. 2020;000:100331. Available froim: https://doi.org/10.1016/j.eclinm.2020.100331
12. Kissler SM, Tedjanto C, Goldstein E, Grad YH, Lipsitch M. Projecting the transmission dynamics of SARS-CoV-2 through the postpandemic period. Science (80-) [Internet]. 2020 Apr 14 [cited 2020 Apr 27];eabb5793. Abailable from: https://www.sciencemag.org/lookup/doi/10.1126/science.abb5793
13. Ferstad JO, Gu AJ, Lee RY, Thapa I, Shin AY, Salomon JA, et al. A model to forecast regional demand for COVID-19 related hospital beds. medRxiv. 2020 Apr 7;2020.03.26.20044842.
14. Orientações sobre Diagnóstico, tratamento e Isolamento de Pacientes com COVID-19. Hessel Dias | Journal of Infection Control [Internet]. [cited 2020 Apr 20]. Available from: https://jic-abih.com.br/index.php/jic/article/view/295
15. Hellewell J, Abbott S, Gimma A, Bosse Ni, Jarvis CI, Russel TW, et al. Feasibility of controlling COVID-19 outbreaks by isolation of cases and contacts. Lancet Glob Heal. 2020.

PARECER CONSUBSTANCIADO DA CONEP

DADOS DO PROJETO DE PESQUISA

Título da Pesquisa: Estudos técnicos de viabilidade para diagnóstico de Covid-19 por teste rápido não invasivo

Área Temática: Equipamentos e dispositivos terapêuticos, novos ou não registrados no País;

Versão: 5

CAAE: 35555720.7.0000.5225

Instituição Proponente: INSTITUTO DE TECNOLOGIA DO PARANA

Patrocinador Principal: Financiamento Próprio
TERA BRASIL COMERCIO DE MATERIAIS MEDICOS E HOSPITALARES LTDA

DADOS DO PARECER

Número do Parecer: 4.234.085

Apresentação do Projeto:

As informações elencadas nos campos "Apresentação do Projeto", "Objetivo da Pesquisa" e "Avaliação dos Riscos e Benefícios" foram retiradas do arquivo Informações Básicas da Pesquisa (PB_INFORMAÇÕES_BÁSICAS_DO_PROJETO_1595122.pdf, de 10/07/2020) e/ou do Projeto Detalhado (TECPAR_TERA_ANALISE_COVIDversaoCEP10jul2020.pdf, de 15/07/2020).

Introdução:

O surto do novo coronavírus, inicialmente denominado 2019-nCoV, assim que descrito em Wuhan China, e agora conhecido como SARS-CoV2, resultou na declaração de estado de pandemia pela OMS em 11 de março de 2020(1). O estado de exceção e ineditismo determinado pela pandemia implicou em grandes transformações sociais, com enorme impacto (imediato e tardio) sobre a saúde pública e privada mundial. No Brasil, o primeiro caso foi detectado em 25 de fevereiro de 2020 e o poder público estabeleceu medidas de prevenção e controle da COVID-19, seguindo critérios epidemiológicos, sanitários e legais(2). É incerto saber qual será o padrão futuro da atividade da COVID-19, mas certamente há grande impacto para o Brasil(2). Entretanto é certo um fato a ser analisado, o caráter dinâmico da evolução, assim como diferenças no seu comportamento regional em nosso país continental, sem mencionar as abissais diferenças regionais na qualidade dos serviços de saúde pública e privada. A distribuição da crise sanitária

não é homogênea e simultânea em todo o planeta. No Brasil, um país de dimensões continentais, a distribuição é díspar assim como o período dos picos de infecção viral. Nesse sentido o poder público tem um papel fundamental na avaliação permanente do comportamento geográfico da disseminação da COVID-19. Por outro lado, a pandemia não impediu a evolução natural de afecções não relacionadas ao novo coronavírus. Os Sistemas de Saúde em todo o mundo estão sendo desafiados, como nunca antes na história recente. Um dilema se estabelece: Como retornar o quotidiano sem acarretar em maiores complicações? Imprescindível destacar que a retomada de forma não estruturada pode resultar em aumento da mortalidade(8–10)(11)Uma forma possível é buscar métodos diagnósticos rápidos de baixo custo e alta efetividade. Caso seja possível encontrar um método que consiga fazer o screening em massa, seria possível retomar atividades com segurança.
Dentro das possibilidades aventadas, estão métodos diagnósticos através de equipamentos de sopro, semelhantes aos bafômetros, que possam detectar diretamente a presença o afirmar a inexistência de vírus SARS-CoV-2. A literatura mundial aponta uma vasta literatura de métodos em desenvolvimento para análise de VOC (Volatile Organic Chemicals) para diagnóstico de pneumonias por variados patógenos. (3–7)A empresa Tera de Israel desenvolveu pesquisas iniciais na área de diagnóstico de SARS-CoV-2 através de análise de sopro (Breath Analysis) com resultados promissores conforme apontados em resultados preliminares. Após acordo internacional entre a empresa Tera de Israel e o Estado do Paraná, foi proposto um protocolo de análise de efetividade da tecnologia de forma prospectiva conforme protocolo anexo.

Hipótese:
Caso seja possível encontrar um método que consiga fazer o screening em massa, seria possível retomar atividades com segurança.

Metodologia Proposta:
Detecção da presença de VOC (Volatile Organic Chemicals) de SARS CoV-2 em amostras colhidas de pacientes testados por características espectrais na banda THz em amostras de ar expirado na membrana de um tubo de ensaio de análises respiratórias. A análise espectral é feita no espectro de 300-3000GHz.O RT-PCR será realizado pelo LACEN ou IBMP de acordo com o protocolo atual será considerado o comparador padrão ouro. O grupo que realizará o PCR está cego aos resultados da Tera Teste de análisede sopro (BAT: Breath Analysis Test) realizado por técnicos cegos para o resultado da PCR.Se o PCR for positivo e o BAT é negativo, o BAT deve ser repetido

assim que o resultado do PCR estiver disponível.
Se o BAT teste for positivo e o PCR for negativo, o PCR deve ser repetido assim que possível.Se tanto BAT quanto PCR são concordantes (negativos ou positivos), nenhum exame precisa ser repetido

Critério de Inclusão:
Masculino e Feminino.
Idade 18 anos.
O paciente deve aceitar e assinar o termo de consentimento livre e esclarecido.
O status de portador do vírus SARS-COV-2 será determinado em uma amostra coletada ao mesmo tempo que o teste de análise da respiração (BAT).
Casos ambulatoriais.
Sintomático e assintomático.

Critério de Exclusão:
Pacientes hospitalizados.
Sujeitos sob anestesia geral.
A incapacidade de assinar pessoalmente o termo de consentimento.
A incapacidade de soprar 5x no teste.Amostras de BAT analisadas após 6h.
Impossibilidade de ter uma segunda amostra.
Pacientes sintomáticos com mais de 5 dias de sintomas para a primeira amostra de PCR ou mais de 7 dias para repetição.

Objetivo da Pesquisa:
Objetivo Primário
Desenvolvimento de um teste rápido, barato e assertivo para descartar SARS CoV-2 em ar expirado.

Objetivos Secundários
Estabelecer o banco de dados preliminar para um estudo de viabilidade rápida. Se for constatado que o vírus pode de fato ser identificado (True positive), ou casos descartados (True negativo), a pesquisa deve ser ampliada para permitir o desenvolvimento rápido de testes de triagem para SARS CoV-2.

Avaliação dos Riscos e Benefícios:

Riscos:

Como os dados preliminares do estudo referem-se a um número pequeno de pacientes, o risco é que em uma amostragem maior esses dados não sejam extrapolados.

Benefícios:

Trata-se da fase de desenvolvimento final de uma aplicação para diagnósticos biológicos de Covid-19. Dados preliminares apontam o potencial de aplicação do método para diagnóstico de pacientes negativos para Covid-19. Alternativa não invasiva para coleta de amostra de respiração. Resposta rápida para pacientes negativos.A validação do método representa uma opção importante na liberação da circulação de pessoas em espaços de grande circulação ou aglomerações.

Comentários e Considerações sobre a Pesquisa:

Detecção da presença de VOC (Volatile Organic Chemicals) de SARS CoV-2 em amostras colhidas de pacientes testados por características espectrais na banda THz em amostras de ar expirado na membrana de um tubo de ensaio de análises respiratórias. A análise espectral é feita no espectro de 300-3000GHz.

O RT-PCR será realizado pelo LACEN ou IBMP de acordo com o protocolo atual será considerado o comparador padrão-ouro. O grupo que realizará o PCR está cego aos resultados da Tera.

Teste de análise de sopro (BAT: Breath Analysis Test) realizado por técnicos cegos para o resultado da PCR.

Se o PCR for positivo e o BAT é negativo, o BAT deve ser repetido assim que o resultado do PCR estiver disponível. Se o BAT teste for positivo e o PCR for negativo, o PCR deve ser repetido assim que possível. Se tanto BAT quanto PCR são concordantes (negativos ou positivos), nenhum exame precisa ser repetido.

1. Grupo 1.1 – PCR sintomático positivo
2. Grupo 1.2 – PCR sintomático negativo
3. Grupo 2.1 – PCR assintomático positivo
4. Grupo 2.2 – PCR assintomático negativo

O estudo está previsto para incluir um mínimo de 1.500 participantes testados verificados como

portadores COVID-19, e um mínimo de 1500 participantes verificados como não-portadores.
Entretanto, haverá uma análise provisória quando 100 PCR positivos foram incluídos. Essa análise fornecerá dados adicionais para ajustar o número total de pacientes a serem testados.

Considerações sobre os Termos de apresentação obrigatória:
Vide campo "Conclusões ou Pendências e Lista de Inadequações".

Recomendações:
Vide campo "Conclusões ou Pendências e Lista de Inadequações".

Conclusões ou Pendências e Lista de Inadequações:
Análise de respostas ao parecer pendente nº 4.219.100 emitido pela Conep em 18/08/2020:

1. Quanto a Folha de Rosto:

1.1. Os documentos submetidos ao Sistema CEP/Conep que necessitam de assinatura dos responsáveis devem ser encaminhados, preferencialmente, com certificação digital ou por documento digitalizado assinado. Será aceita, em caráter excepcional, a dispensa das assinaturas nos documentos necessários para a submissão dos protocolos de pesquisa, durante o período em que estiverem instaladas as medidas de emergência sanitária decorrente da pandemia COVID-19. Recomenda-se que posteriormente sejam encaminhados, via NOTIFICAÇÃO, todos os documento e declarações pertinentes ao estudo em tela, devidamente preenchidos e assinados. Contudo, é indispensável a assinatura do campo Pesquisador Responsável. Solicita-se adequação.
RESPOSTA: Os documentos que necessitam de assinatura dos responsáveis foram adequados e, portanto, estamos submetendo o documento digitalizado assinado.
ANÁLISE: PENDÊNCIA ATENDIDA.

1.2. No documento Projeto Detalhado, o pesquisador informa que uma parte dos custos da pesquisa destina -se a empresa Terra Brasil. Caso o patrocinador não seja o pesquisador responsável, solicita-se que seja inserida na Plataforma Brasil nova Folha de Rosto com o campo patrocinador devidamente preenchido, datado e assinado (salienta-se que nos casos de agências de fomento não é necessária a assinatura no campo referente ao patrocinador). Solicita-se, adicionalmente, que os dados relacionados ao patrocinador sejam corrigidos na Plataforma Brasil (Item "Financiamento", Aba 3 "Desenho de Estudo/ Apoio Financeiro").
RESPOSTA: A folha de rosto foi alterada, constando, portanto, a assinatura do representante da

patrocinadora principal após inserção no item “financiamento da aba 3”.
ANÁLISE: PENDÊNCIA ATENDIDA.

2. Quanto ao Projeto Detalhado, documento intitulado "TECPAR_TERA_ANALISE.pdf":

2.1. Solicitam-se esclarecimentos quanto a Participação da empresa Terra de Israel no estudo.
RESPOSTA: No projeto detalhado a participação da equipe da Tera Group LTD de Israel foi mais bem detalhada.
ANÁLISE: PENDÊNCIA ATENDIDA.

3. Quanto ao Termo de Consentimento Livre e Esclarecido, documento intitulado "TCLE.pdf":

3.1. Na primeira página, lê-se: "Esta PESQUISA é considerada o padrão ouro, ou seja, o melhor método diagnóstico existente." (Destaque nosso). Entende-se que o termo destacado faz menção ao teste de swab (técnica RT-PCR). Para o melhor esclarecimento do participante da pesquisa, solicita-se substituir o termo em destaque.
RESPOSTA: Esclarecido no TCLE que o padrão ouro se refere ao RT-PCR.
ANÁLISE: PENDÊNCIA ATENDIDA.

3.2. Na segunda página, lê-se: "A sua participação neste estudo é voluntária e se o Sr/Sra não quiser mais fazer parte da pesquisa, poderá desistir a qualquer momento e solicitar que lhe devolvam este Termo de Consentimento Livre e Esclarecido assinado.". Deve ser informado que o TCLE será elaborado em duas vias, sendo uma retida com o pesquisador responsável e outra com o participante de pesquisa, conforme Resolução CNS Nº 466 de 2012 itens IV.3.f e IV.5.d.
RESPOSTA: Esclarecido que o TCLE será gerado em duas vias, ficando uma com o paciente.
ANÁLISE: PENDÊNCIA ATENDIDA.

3.3. Deve ser garantido ao participante de pesquisa e seu acompanhante o ressarcimento de despesas decorrentes da participação no estudo nos dias em que for necessária sua presença para consultas ou exames. Sendo assim, solicita-se garantir, de forma clara e afirmativa, o ressarcimento das despesas tidas pelo participante da pesquisa e de seu acompanhante, se necessário, em decorrência de sua participação na pesquisa, podendo-se citar como exemplo, o transporte e a alimentação, mas não se restringindo a eles (Resolução CNS nº 466 de 2012, itens

II.21 e IV.3.g).
RESPOSTA: Esclarecido no TCLE que o paciente terá o ressarcimento de qualquer despesa relacionada a essa pesquisa.
ANÁLISE: PENDÊNCIA ATENDIDA.

3.4. Solicita-se que seja expresso, de modo claro e afirmativo no TCLE, o direito a assistência integral gratuita devido a danos diretos/ indiretos e imediatos/ tardios, pelo tempo que for necessário ao participante da pesquisa (Resolução CNS nº 466 de 2012, itens II.3.1 e II.3.2).
RESPOSTA: Esclarecido no TCLE que o paciente terá assistência integral pelo tempo que for necessário.
ANÁLISE: PENDÊNCIA ATENDIDA.

Considerações Finais a critério da CONEP:
Diante do exposto, a Comissão Nacional de Ética em Pesquisa - Conep, de acordo com as atribuições definidas na Resolução CNS nº 466 de 2012 e na Norma Operacional nº 001 de 2013 do CNS, manifesta-se pela aprovação do projeto de pesquisa proposto.

Situação: Protocolo aprovado.

BRASILIA, 25 de Agosto de 2020

____
