# Supplementary material for: Clinical trial and detection of SARS-CoV-2 by a commercial breath analysis test based on Terahertz technology
Source: PLoS One. 2022 Sep 20;17(9):e0273506. doi: 10.1371/journal.pone.0273506 (PMC9488804; doi:10.1371/journal.pone.0273506)
Supplement: Attachment — Submitted filename: Rebuttal letter_BreathTerahertz _03rdJun2022.docx [file pone.0273506.s004.docx]

Curitiba, Jun 03^rd^, 2022

**Response letter**

To: Davor Plavec, MD, MSc, PhD, Prof.

Academic Editor

Re: PLOS ONE Decision: Revision required [PONE-D-22-07738]

Dear Dr Plavec,

Please find attached our reviewed manuscript entitled “Clinical trial and detection of SARS-CoV-2 by a commercial breath analysis test based on Terahertz technology” [PONE-D-22-07738]. All the corrections and suggestions indicated by the reviewers were fully addressed, accepted, and rewritten.

A response letter has been added to pinpoint the editor and reviewers’ requests, with a detailed list of corrections and a description of changes made within the manuscript. To better identify the corrections of each reviewer, changes were highlighted in different colors in the revised manuscript. Thank you for the opportunity and please do not hesitate to contact us with any further question.

Sincerely,

Alexander Welker Biondo, DMV, MSc, PhD.

Professor, Department of Veterinary Medicine, UFPR, Brazil

Visiting Professor, Purdue University, IN, USA

E-mail: [abiondo@ufpr.br](about:blank) / Phone: +55 (41) 3350-5812

**Manuscript #PONE-D-22-07738**

**Title** “Clinical trial and detection of SARS-CoV-2 by a commercial breath analysis test based on Terahertz technology”

1. Thank you for stating the following Funding Information in your manuscript:

"Brazilian National Council for Scientific and Technological Development -CNPq (material support) (2020-1/402341- AWB and CPB). The funders had no role in study design, data collection and analysis, decision to publish, or preparation of the manuscript."

We note that you have provided funding information that is currently declared in your Funding Statement. However, funding information should not appear in any areas of your manuscript. We will only publish funding information present in the Funding Statement section of the online submission form.

Please remove any funding-related text from the manuscript.

**Answer:** Funding information was removed from the manuscript.

**Review Comments to the Author**

Please use the space provided to explain your answers to the questions above. You may also include additional comments for the author, including concerns about dual publication, research ethics, or publication ethics. (Please upload your review as an attachment if it exceeds 20,000 characters)

Reviewer #1: Interesting results that should be tested in the broader population and different circumstances, but this work is pointing in the direction that this system can be used in Covid 19 pandemic and maybe for the further possible epidemics. The test for discrimination between the respiratory infection and specific respiratory infection is warranted.

**Answer:** Authors are thankful to reviewer for the positive comment.

Reviewer #2: In the paper authors presented results of clinical trial in which evaluate a commercial breath analysis test (TERA.Bio®) for detecting the SARS-CoV-2 present in exhaled air samples of suspicious persons. All components of the manuscript are correctly presented, including introduction, results, statistical analysis, discussion and conclusions.

**Answer:** Authors are thankful to reviewer for the positive comment.

However, the study conducted has certain limitations;

1. the potential bias of symptomatic cases which may have partially compromised the assessment of breath analysis thest (BAT) for asymptomatic patients

2. the BAT did not tested in asymptomatic patients and patients in the first days of infection or after 7 days of the onset of symptoms

3. the small sample size of infected persons (70 confirmed infected persons)

4.relatively limited detection of occurrence of other respiratory viruses, or other pathogens (including bacterial), for evidence of co-infection in patients with COVID-19 .

All these limitations were anticipated by the authors and suggested how to improve them.

**Answer:** Authors are thankful to reviewer for the positive comment, since all above limitations have already been reported and discussed intext.

Reviewer #3 (green intext): This is a well-written paper with innovations in SARS-COV2 diagnostics. In the Materials and Methods section, it would be good to explain the detailed procedure as it relates to the collection of exhaled air samples to prevent the spread of SARS-CoV2.

**Changed:** Reviewer is right, and information was added.

**Now you read:** “The study protocol has included non-hospitalized patients who were referred by the Curitiba city health professionals after suspicious RT-qPCR results. Thus, exhaled air samples and nasopharyngeal swab samples were concomitantly collected and tested by the official city service. Each patient sampled by nasopharyngeal swab was also given a dischargeable kit of exhaled air analyzer containing an identified sampling device, which was uncovered in both sides with opened capsule and the melamine membrane internally coupled. Patients were then asked to keep a 2-meter distance from the operator, swallow all saliva in the mouth and deeply blow the device for 5 times. After blown, the patient was asked to close both device sides with the correspondent lids (within the kit) until hear a click noise of sealed lid, capturing the exhaled breath constituents inside the device. The operator received the device, inspected the complete lid closure, and disinfected the external parts with 70% alcohol. After taken to the laboratory, device was opened and capsule with internal membrane removed and inserted into the equipment for analysis. Samples were collected up to 6 hours maximum after sampling, which insured best readings due to membrane integrity, ideal absorption of electromagnetic wave and wave propagation into the exhaled air sample. After analysis, device was discharged as hospital contaminated garbage.” (Page 9-10, Lines 238-258).

It is visible that it is done in disposable air collection kit, but can it be sampled next to the patient (since these are all outpatient samples, not from hospitalized patients). What about safety to the environment during the sampling? Does the time between sampling and analysis influences the results?

**Changed:** Reviewer is right, and information was added.

**Now you read:** “The study protocol has included non-hospitalized patients who were referred by the Curitiba city health professionals after suspicious RT-qPCR results. Thus, exhaled air samples and nasopharyngeal swab samples were concomitantly collected and tested by the official city service. Each patient sampled by nasopharyngeal swab was also given a dischargeable kit of exhaled air analyzer containing an identified sampling device, which was uncovered in both sides with opened capsule and the melamine membrane internally coupled. Patients were then asked to keep a 2-meter distance from the operator, swallow all saliva in the mouth and deeply blow the device for 5 times. After blown, the patient was asked to close both device sides with the correspondent lids (within the kit) until hear a click noise of sealed lid, capturing the exhaled breath constituents inside the device. The operator received the device, inspected the complete lid closure, and disinfected the external parts with 70% alcohol. After taken to the laboratory, device was opened and capsule with internal membrane removed and inserted into the equipment for analysis. Samples were collected up to 6 hours maximum after sampling, which insured best readings due to membrane integrity, ideal absorption of electromagnetic wave and wave propagation into the exhaled air sample. After analysis, device was discharged as hospital contaminated garbage.” (Page 9-10, Lines 238-258).
