# Supplementary material for: Clinical trial and detection of SARS-CoV-2 by a commercial breath analysis test based on Terahertz technology
Source: PLoS One. 2022 Sep 20;17(9):e0273506. doi: 10.1371/journal.pone.0273506 (PMC9488804; doi:10.1371/journal.pone.0273506)
Supplement: Attachment — Submitted filename: Response letter_BreathTerahertz _01stJul2022 final.docx [file pone.0273506.s005.docx]

Curitiba, July 1^st^, 2022

**Response letter**

To: Davor Plavec, MD, MSc, PhD, Prof.

Academic Editor

Re: PLOS ONE Decision: Revision required [PONE-D-22-07738R1]

Dear Dr Plavec,

Please find attached our reviewed manuscript entitled “Clinical trial and detection of SARS-CoV-2 by a commercial breath analysis test based on Terahertz technology” [PONE-D-22-07738R1]. All the corrections and suggestions indicated by the reviewers were fully addressed, accepted, and rewritten.

A response letter has been added to pinpoint the editor and reviewers’ requests, with a detailed list of corrections and a description of changes made within the manuscript. To better identify the corrections of each reviewer, changes were highlighted in different colors in the revised manuscript. Thank you for the opportunity and please do not hesitate to contact us with any further question.

Sincerely,

Alexander Welker Biondo, DMV, MSc, PhD.

Professor, Department of Veterinary Medicine, UFPR, Brazil

Visiting Professor, Purdue University, IN, USA

E-mail: [abiondo@ufpr.br](about:blank) / Phone: +55 (41) 3350-5812

**Manuscript #PONE-D-22-07738R1**

**Title** “Clinical trial and detection of SARS-CoV-2 by a commercial breath analysis test based on Terahertz technology”

Reviewer #4: A clinical trial was conducted which aimed to evaluate the commercial breath analysis test TERA.Bio (r) and a deterministic algorithm for detecting SARS-CoV-2 compounds exhaled in air samples. Results were compared to RT-qPCR as the gold standard. The sensitivity and specificity of the test was 92.6% and 96.0%, respectively.

Minor revisions:

1- State and justify the study’s target sample size with a pre-study statistical power calculation.

**Changed:** Reviewer is right, that information was missing. Explanation was included in the Study Sample Size and Data Validation Section of the manuscript. The correspondent reference was cited in the sentence.

**Now you read:** “The assumed positivity of RT-qPCR for sampling calculation in the clinical trial herein was 25%, based on epidemiological COVID-19 reports of Curitiba city, which had an estimated population of approximately 1.9 million habitants at the time. Aiming at a type I error (α) of 0.05, and an estimated precision of 0.05, a minimum sample size of 400 subjects was obtained to statistically evaluate the BAT test, considering a 90% sensitivity as minimum for a useful diagnostic test (30). Representing a consecutive, casuistic, random, and homogeneous sampling, collected from all subjects who presented themselves at the hospital in the period, a total of 570 successful samplings were included in the present study.” (Page 7-8, Lines 173-183).

2- In the statistical analysis section indicate the methods used to compare the features listed in table 1. Provide more precise p-values rather than p < 0.05.

**Changed:** The sentences were included in the Statistical Analysis section of the manuscript.

P values were included, whenever higher than 0.0001. P values lower than 0.0001 are not expressed by the software used for statistical analysis (SPSS).

**Now you read:** “Sociodemographic, epidemiological, and clinical characteristics were presented as percentages, alongside their 95% proportion confidence intervals, and by arithmetic mean and standard deviation. The results were presented in contingency tables, allowing the calculation of sensitivity, specificity, and predictive values for the BAT. Assessment of the association between categorical variables; and the BATs and RT-qPCR tests was performed by Fisher’s exact test. Continuous variables were compared using Student’s unpaired t-test. The precision of the BAT was assessed using ROC curve analysis. Statistical significance was assumed for P<0.05.

In addition, machine learning was based on “training data” that had been collected prior to the study herein. Over 4,000 subjects were tested using the BATs at several locations worldwide including Brazil. The THz spectrum features for healthy and infected (negative and positive) subjects were processed using ML techniques, to establish a mathematical algorithm for COVID-19 classification. The statistical analyses were performed using the SPSS version 17 software.” (Page 11-12, Lines 290-306).

3- Lines 364-370 and subsequent sections: In the statistical analysis section, state the method used to calculate the confidence intervals.

**Changed:** The information was included in the Statistical analysis Section of the manuscript.

**Now you read: “**The results from the RT-qPCR test and SARS-CoV-2 BAT for all subjects was presented (Table 2). Using RT-qPCR method as the gold standard, the commercial BAT method was found to have 92.7% sensitivity (CI 84.1-97.6%) and 96.0% specificity (CI 93.9-97.5%). The positive predictive value (PPV) and negative predictive value (NPV) were determined as 76.5% (CI 66.3-85.0%) and 99.0% (CI 97.6-99.7%), respectively. Fisher's exact test showed a statistically significant association between results (P < 0.0001). The area under the ROC curve of the total sampling was 0.94 (SD 0.19; CI 0.91-0.98) (Figure 4).

**Table 2.** Results from RT-qPCR and BAT for the entire sample.

|  |  | RT-qPCR SARS-CoV-2 results | | |
| --- | --- | --- | --- | --- |
|  |  | Positive (%) | Negative (%) | Total (%) |
| Results  COVID-19 BAT test | Positive | 65 (11.4) | 20 (3.5) | 85 (14.9) |
|  | Negative | 5 (0.9) | 480 (84.2) | 485 (85.1) |
|  | Total | 70 (12.3) | 500 (87.7) | 570 (100.0) |

Figure 4. ROC curve for the entire sample.

Considering only the symptomatic patients and using RT-qPCR as the gold standard, BAT presented 92.4% sensitivity (CI 83.2-97.5%) and 95.9% specificity (CI 93.1-97.7%). The PPV was determined as 81.3% (CI 70.7-89.4%) and the NPV as 98.5% (CI 96.5-99.5%). Fisher's exact test showed a statistically significant association between results (p<0.0001). The area under the ROC curve calculated only with symptomatic patients was 0.94 (standard error 0.20; CI 0.90-0.98).

Given that the optimum sampling window of opportunity for RT-qPCR has been reported to comprise the first seven days with symptoms, the data were stratified for analysis including the symptomatic patients who had shown symptoms for up to seven days. In such scenario, the results from SARS-CoV-2 BAT, compared with the RT-qPCR method as the gold standard, showed 90.2% sensitivity (CI 76.0-97.3%) and 95.5% specificity (CI 90.9-98.2%). The PPV was determined as 84.1% (CI 69.9-93.4%) and the NPV as 97.4% (CI 93.4-99.3%). Again, Fisher's exact test showed a statistically significant association between the results obtained through the two methods (P < 0.0001). The area under the ROC curve calculated only for symptomatic patients who had shown symptoms up to seven days was 0.93 (standard error 0.03; CI 0.87-0.98).

Considering only the asymptomatic patients, the results showed 100.0% sensitivity (CI 39.7-100.0%) and 96.3% specificity (CI 92.1- 98.6%), with PPV of 40.0% (CI 12.2-73.8%) and NPV of 100.0% (CI 97.7-100.0%). Fisher's exact test showed a statistically significant association between results (P < 0.0001). The area under the ROC curve calculated only with asymptomatic patients was 0.98 (standard error 0.01; CI 0.96-1.00). The limitation of this method was a false positive rate of 23.5%, indicating that the positive predictive value of the test may be compromised in this sampling.” (Page 15-17, Lines 358-401).

4- Table 2: In addition to frequencies, provide the corresponding percentages.

**Changed:** The percentages were included in Table 2.

**Now you read:**

**Table 2.** Results from RT-qPCR and BAT for the entire sample.

|  |  | RT-qPCR SARS-CoV-2 results | | |
| --- | --- | --- | --- | --- |
|  |  | Positive (%) | Negative (%) | Total (%) |
| Results  COVID-19 BAT test | Positive | 65 (11.4) | 20 (3.5) | 85 (14.9) |
|  | Negative | 5 (0.9) | 480 (84.2) | 485 (85.1) |
|  | Total | 70 (12.3) | 500 (87.7) | 570 (100.0) |
