# Supplementary material for: Clinical trial and detection of SARS-CoV-2 by a commercial breath analysis test based on Terahertz technology
Source: PLoS One. 2022 Sep 20;17(9):e0273506. doi: 10.1371/journal.pone.0273506 (PMC9488804; doi:10.1371/journal.pone.0273506)
Supplement: Attachment — Submitted filename: Response to reviewers_BreathTerahertz _16thJun2022.docx [file pone.0273506.s006.docx]

Curitiba, July 11^th^, 2022

**Response letter**

To: Davor Plavec, MD, MSc, PhD, Prof.

Academic Editor

Re: PLOS ONE Decision: Revision required [PONE-D-22-07738R2]

Dear Dr Plavec,

Please find attached our reviewed manuscript entitled “Clinical trial and detection of SARS-CoV-2 by a commercial breath analysis test based on Terahertz technology” [PONE-D-22-07738R1]. All the corrections and suggestions indicated by the reviewers were fully addressed, accepted, and rewritten.

A response letter has been added to pinpoint the editor and reviewers’ requests, with a detailed list of corrections and a description of changes made within the manuscript. To better identify the corrections of each reviewer, changes were highlighted in different colors in the revised manuscript. Thank you for the opportunity and please do not hesitate to contact us with any further question.

Alexander Welker Biondo, DMV, MSc, PhD.

Professor, Department of Veterinary Medicine, UFPR, Brazil

Visiting Professor, Purdue University, IN, USA

E-mail: [abiondo@ufpr.br](about:blank) / Phone: +55 (41) 3350-5812

**Manuscript #PONE-D-22-07738R1**

**Title** “Clinical trial and detection of SARS-CoV-2 by a commercial breath analysis test based on Terahertz technology”

Reviewer #4: Minor revisions: (Note that line numbers refer to those in the track changes version of the manuscript.)

1- Line 183: In the sample size justification section, state the statistical power that was attained.

**Changed:** The statistical power was included.

**Now you read: “**Aiming at a type I error (α) of 0.05, and an estimated precision of 0.05, a minimum sample size of 400 subjects was obtained to statistically evaluate the BAT test, considering a 90% sensitivity as minimum for a useful diagnostic test (30). Representing a consecutive, casuistic, random, and homogeneous sampling, collected from all subjects who presented themselves at the hospital in the period, a total of 570 successful samplings were included in the present study, with statistical power of 0.819.” (Page 8, Lines 193).

2- Table 1: Some of the p-values display a comma in place of a decimal. Perhaps these errors resulted when the document was formatted by the journal.

**Changed:** Number have been corrected at table 1.

**Now you read:**

**Table 1.** Sociodemographic, epidemiological, and clinical data of the 570 subjects included in the present study.

| Features* | Confirmed cases (n=70) | Unconfirmed cases (n=500) | P-value^a^ |
| --- | --- | --- | --- |
| Age (years) | 38.6 ±11.1 | 37.1±12.5 | 0.345 |
| Males | 41 (58.0%) | 233 (46.6%) | 0.060 |
| Body mass index (kg/m^2^) | 27.6 ± 4.7 ^d^ | 25.9 ± 4.5 | 0.004 |
| Non-white race | 25 (40.3%) ^a^ | 71 (15.4%) | 0.0001 |
| Travel history | 07 (10%) | 81 (16.7%) | 0.109 |
| Suspicious case contact | 35 (50%) | 218 (43.6%) | 0.232 |
| Confirmed case contact | 32 (45.7%) ^a^ | 156 (31.2%) | 0.026 |
| Attended at health service | 20 (28.6%) | 185 (38.2%) | 0.090 |
| Symptomatic | 66 (94.3%) ^a^ | 338 (67.6%) | 0.0001 |
| Days of symptoms ^b^ | 5.4 ± 2.6 (75.7%) | 5.1 ± 3.4 (36.4%) | 0.468 |
| Fever or chills | 55 (78.6%) ^a^ | 93 (18.6%) | 0.0001 |
| Myalgia or arthralgia | 63 (90.0%) ^a^ | 129 (25.8%) | 0.0001 |
| Headache | 44 (62.9%) ^a^ | 218 (44.9%) | 0.005 |
| Respiratory symptoms ^c^ | 65 (92.9%) ^a^ | 261 (52.2%) | 0.0001 |
| Gastrointestinal symptoms ^d^ | 38 (54.3%) ^a^ | 71 (14.2%) | 0.0001 |
| Anosmia | 34 (48.6%) ^a^ | 42 (8.7%) | 0.0001 |
| Ageusia | 35 (50.0%) ^a^ | 37 (7.6%) | 0.0001 |
| Rash | 4 (6.1%) | 12 (2.5%) | 0.987 |
| Smoking | 4 (5.7%) ^a^ | 67 (13.4%) | 0.05 |
| Pneumopathy | 7 (10%) | 43 (8.6%) | 0.698 |
| Diabetes | 3 (4.3%) | 12.0 (2.4%) | 0.214 |
| Hypertension | 5 (7.1%) | 43 (8.6%) | 0.280 |
| Other comorbidities | 2 (2.9%) ^a^ | 04 (0.8%) | 0.007 |

^a^p<0.05. Continuous variables are presented as mean and standard deviation. ^b^Date of onset of symptoms was not reported by the patients. ^c^ Coughing, odynophagia, dyspnea, nasal drainage. ^d^ vomiting, diarrhea.

3- In the "Statistical analysis" section, list and describe the statistical methods used to estimate the p-values shown in Table 1. Also indicate the statistical methods used to generate 95% confidence intervals.

**Changed:** Statistical methods were inserted.

**Now you read: “**Student's t-test was used to determine estimate the p-values and chi-square test to generate 95% confidence intervals.” (Page 11, Lines 299-301).
